# Mesotherapy in Management of Hairloss — Is it of Any Use?

**DOI:** 10.4103/0974-7753.66914

**Published:** 2010

**Authors:** Venkataram Mysore

**Affiliations:** Center for Advanced Dermatology, Bangalore, India

**Keywords:** Mesotherapy, hair loss, androgenetic alopecia

## Abstract

Mesotherapy has received a lot of publicity in the media and internet about its possible role in androgenetic alopecia. However, the subject is controversial in view of lack of documented evidence. This article provides a critical commentary on the use of mesotherapy in the management of androgenetic alopecia.

Mesotherapy, a technique involving injections of medications directly into the skin, was first described by a French physician, Dr. Michel Pistor.[1] He first administered procaine intravenously to treat an asthmatic patient and found that the patient’s hearing improved. He then started experimenting on superficial injections of procaine for various indications and coined the term “mesotherapy” in 1976. It has been subsequently tried for treatment of many other conditions such as joint pain, eczema and tinnitus.[2,3] The term mesotherapy was coined to denote “treatment of the mesoderm”.[4]

Mesotherapy has recently attracted interest, curiosity and publicity as a treatment modality for several cosmetic indications such as cellulite, aging skin and body contouring.[5–8] There have been claims and reports in internet sites and lay press about its use in management of hairloss too. In India, several esthetic, beauty and hair clinics (some of which are run by nonprofessionals) have advertised this modality extensively in lay press, as a safe and effective remedy for androgenetic alopecia, leading to many patients seeking this treatment. Dermatologists are frequently sought opinion as to the efficacy of such treatments. A critical analysis is therefore necessary to ascertain the role of this treatment in pattern hairloss, if any.

What strikes the author while searching for documented material on the subject, is the near total lack of documented data about all aspects of the treatment — mechanism of action, pharmacokinetics, pharmacodynamics, therapeutic regimes, etc., in standard medical journals. Inhouse publications and company brochures suggest that injections of “cocktails” of natural plant extracts, homoeopathic agents, vasodilators, finasteride and minoxidil and vitamins are carried out with a mesogun once in 2—4 weeks. Of these, only minoxidil and finasteride have an established role in the management of pattern hairloss. Both the mechanism of action and the efficacy of other agents are not established and are doubtful.

It is interesting to note that while medline search for key words “mesotherapy” and “hairloss” reveals only two results, a similar search in google reveals 209,000 results!!! Also, the two results on medline search are reports of side effects following the use of mesotherapy for hairloss! One report documented the development of alopecia in two patients following the use of mesotherapy for hairloss.[9] Histopathologic features showed an almost complete absence of terminal anagen hair follicles and a marked increase in the number of telogen germinal units and catagen follicles, suggesting acute noncicatricial alopecia similar to anagen effluvium. The pathogenesis of hair loss in both the cases was difficult to establish. The other article reports the development of multiple abscesses,[10] thereby suggesting that the treatment could have serious adverse effects.

It is also relevant to note here that Federal drug administration (FDA), USA, has not approved this method of treatment for any indication. The CDC has recommended that “providers should adhere to recommend standard precautions, follow safe-injection practices with appropriate aseptic techniques, and inject only FDA approved products that are prepared following guidelines to ensure sterility as described in the FDA’s good manufacturing practices.”[4]

Further, a recent publication of guidelines on esthetic practices in Singapore listed mesotherapy as a List B procedure (List B procedure indicates procedures with low or very low evidence or local medical expert consensus that *procedure is neither well-established nor acceptable.*)[11]

In view of these data, the current position on the use of mesotherapy in pattern hairloss can be summarized as follows.

1. Data on its safety and efficacy in pattern hairloss have not been adequately and critically evaluated and documented in proper, peer-reviewed clinical trials.
2. Data evaluating the rationale and pharmacology of the combination of herbal and allopathic medicines used are not adequate. There are no clear-cut guidelines on the dosage and efficacy of the products.
3. Further, mesotherapy is not entirely a safe technique as publicized in lay media and can give rise to complications, as stated earlier.

Routine use of the technique is therefore not justified. Well-designed controlled scientific studies are required to substantiate the claims of effectiveness of these products. Regulatory authorities and associations have a role to play in ensuring such studies. What is also needed is proper public health education to ensure quality, ethical management and prevention of commercial exploitation of patients.
